# Prevalence of pulmonary hypertension in children with obstructive sleep apnea living at high altitude

**DOI:** 10.1016/j.sleepx.2024.100106

**Published:** 2024-02-02

**Authors:** Elida Duenas-Meza, Diego Fernando Severiche-Bueno, Carolina Santos Quintero, Jenny Talani Ochoa, Miguel Ronderos Dummit, Claudia Stapper, Carlos Granados G

**Affiliations:** a Fundación Neumológica Colombiana, Bogotá, Colombia; b Departamento de Pediatría, Universidad de La Sabana, Chía, Cundinamarca, Colombia; c Fundación CardioInfantil, Bogotá, Colombia

**Keywords:** Obstructive sleep apnea, Pulmonary hypertension, Prevalence, Children, High altitude

## Abstract

**Introduction:**

The prevalence of obstructive sleep apnea (OSA) is 1–4 %. Some reports describe its association with pulmonary hypertension (PH), but its prevalence is unknown. No studies at high altitude have determined the relationship between OSA and PH. The aim of this study was to establish the prevalence of PH in children diagnosed with OSA living in a high-altitude city at 2640 m above sea level.

**Methods:**

Children between 2 and 16 years of age referred to the Sleep Laboratory of the Fundación Neumológica Colombiana in Bogotá with a positive polysomnogram for OSA were included, and a two-dimensional transthoracic echocardiogram (TTE) was performed to evaluate PH. Statistical analysis was performed using median, interquartile range, chi-squared test, and Kruskall-Wallis test.

**Results:**

Of the 55 patients (n: 55), 63.6 % were male, with a median age of 6 years, 14 children (25.5 %) were overweight; 12 children (21.8 %) had mild OSA, 12 (21.8 %) had moderate OSA and 31 (56.4 %) severe OSA. In patients with severe OSA, the minimum saturation during events was 78 % with a desaturation index (DI) of 33.8/hour (p < 0.01). T90 and T85 increased proportionally with OSA severity (p < 0.05). Of the 55 patients with OSA, none had PH according to echocardiography; 4 patients (7.2 %) had pulmonary artery systolic pressure (PASP) at the upper limit of normal (ULN), and it was not related to a higher body mass index (BMI).

**Conclusions:**

We found no association between OSA and PH in children with OSA at high altitude.

## Highlights

• The prevalence of pulmonary hypertension in children with OSA living at high altitude is unknown.
• It is not known whether living at high altitude is an additional risk factor for the development of PH in children with OSA.
• None of the children with sleep apnea in this study who lived in a high-altitude city had a PASP that was higher than normal.
• Intermittent desaturation and OSA severity at high altitude do not correlate with development of pulmonary hypertension.
• It is important to identify the subgroups of children with sleep apnea who need to be screened for pulmonary hypertension.

## Abbreviations

OSA: obstructive sleep apnea
TTE: transthoracic echocardiogram
PASP: pulmonary artery systolic pressure
ULN: upper limit of normal
BMI: body mass index
PH: pulmonary hypertension
PSG: polysomnogram
SpO2: Oxygen saturation
AHI: Apnea-Hypopnea Index
TRV: tricuspid regurgitation velocity
HAPH: High-altitude pulmonary hypertension

## 1 Introduction

The prevalence of obstructive sleep apnea (OSA) in children ranges from 1 % to 6 % [1]. Evidence on the cardiovascular effects of OSA in children is limited compared to that in adults, but it may be associated with increased blood pressure, pulmonary hypertension, endothelial dysfunction, and changes in ventricular structure and function [2]. This may be explained by increased sympathetic activity due to frequent arousals and intermittent hypoxia, chronic inflammation due to elevated levels of inflammatory interleukins, and endothelial dysfunction secondary to oxidative stress on blood vessels due to intermittent hypoxia [[2], [3], [4], [5]].

Data on pediatric pulmonary hypertension (PH) are mainly derived from registry cohorts. The estimated prevalence in all categories has been reported to be 20–40 cases per million in Europe and 26–33 million children in the USA [6]. Most cases were transient pulmonary arterial hypertension (PAH) due to persistent PH of the newborn or repairable cardiac shunt defects followed by other forms of PAH (associated with connective tissue disease and pulmonary veno-occlusive disease) and pulmonary hypertension associated with developmental lung disease [6,7].

PH due to lung disease and/or hypoxia is an important and increasingly recognized category in which 10–12 % of children with PH have associated lung disease, particularly developmental lung disease with bronchopulmonary dysplasia being the most common disorder [6,8,9]. The estimated prevalence of PH in children with OSA varies widely. A 2019 study reported a low prevalence (3 %) [10]. However, a more recent study reported a high prevalence (8 %) in patients with severe OSA and echocardiographic evidence of PH. Nonetheless, in this study, 46 % of the children PH had Down syndrome [11].

The article by Peñaloza and Arias describes a study in Morococha, Peru, at an altitude of 4540 m, with a group of 32 children between 1 and 14 years of age [12]. This group underwent cardiac catheterization, and it was observed that mild pulmonary hypertension and increased pulmonary vascular resistance were present and were greater in children under 5 years of age than in those between 6 and 14 years of age [12]. This may be secondary to an increase in the number of smooth muscle cells. However, the same article mentions that these findings seem to be more common in populations living at altitudes above 3000 m [12]. In addition, a study conducted in China among children aged 0–14 years living in Jiuzhi Qinghai (3700 m above sea level), Xining Qinghai (2260 m above sea level), and Shanghai (16 m above sea level) showed that median pulmonary artery pressure (mPAP), as determined by a multiple regression equation, was higher in children living at 3700 m than in those living at 2260 m and 16 m above sea level [13].

It is known that approximately 81.6 million people live permanently at high altitude [14] nevertheless, no studies at high altitude have established the relationship between OSA and PH. Describing the behavior of PH in OSA under these geographic conditions is essential to look for differences in diagnostic approach and management and could apply to millions of children living under the same conditions in Latin America, Asia, and Africa. The objective of this study was to determine the prevalence of PH in children aged 2–16 years old with a diagnosis of OSA living in a high-altitude city located at 2640 m above sea level.

## 2 Methods

An observational cross-sectional study was conducted in children aged 2–16 years who were referred to the Sleep Laboratory of the Fundación Neumológica Colombiana in Bogotá, Colombia, between the months of January 2013 and September 2014 for polysomnography because of clinical suspicion of OSA and whose polysomnogram (PSG) confirmed the disease. All patients had to be residents at an altitude between 2640 and 3000 m above sea level. Patients with prematurity, cardiac disease of any type, genetic syndromes, chronic lung disease, bronchopulmonary dysplasia, cystic fibrosis, severe craniofacial malformations, patients with a previous diagnosis of PH secondary to other causes, neuromuscular diseases, or who did not agree to participate in the study were excluded. All children underwent a complete medical history by a sleep-trained pediatrician and a two-dimensional transthoracic echocardiogram (TTE), and it was a criterion for inclusion that the child's parent or guardian signed the informed consent form. The study was submitted to and approved by the Ethics Committee of the Fundación Neumológica Colombiana.

### 2.1 Polysomnogram

The Pediatric Sleep Questionnaire (PSQ) [15] was administered to all patients, followed by a PSG according to the recommendations of the American Academy of Sleep Medicine (AASM) [16] using a diagnostic sleep system (Philips Respironics®; Alice 5 and LE models). PSG was performed at night for a minimum duration of 6 h, in a quiet environment, with an average temperature of 19 °C. One of the parents accompanied the child during the PSG.

The following parameters were measured: heart rate by electrocardiogram, thoracoabdominal movements by inductance plethysmography, and airflow monitored by nasal pressure cannula and oronasal thermistor. Oxygen saturation (SpO2) levels was monitored with a high-precision oximeter (Massimo®, Massimo Corporation, model Rad 8, Irvine, CA) with simultaneous pulse wave recording integrated into the polysomnograph. Bilateral electrooculogram, electromyogram in the chin and anterior tibial region, three electroencephalogram channels, and 2 sensors, one for body position and another for detection of snoring sounds, were also monitored. A time-synchronized video recording was also obtained.

A sleep technician performed the staging of all the studies, which were reviewed by a pediatric pulmonologist with expertise in sleep medicine. Manual analysis of the recordings was performed according to AASM staging standards [16]. For the analysis of the respiratory variables, we used the following definitions according to the AASM guidelines: Sleep apnea-hypopnea index (AHI) was defined as the number of apneas and hypopneas per hour of total sleep time (TST), apnea if all of the criteria established by the AASSM were met (a decreased in the peak signal excursion by ≥ 90 % of the pre-event baseline using an oronasal thermal sensor, the duration of the ≥90 % decreased in sensor signal lasted at least the minimum duration as specified by the criteria for obstructive, mixed or central sleep apnea duration and the event met the respiratory effort criteria), obstructive apnea if it meets the apnea criteria for at least the duration of 2 breaths during baseline breathing and is associated with the presence of respiratory effort throughout the entire period of absence of airflow and central apnea if it meets apnea criteria and is associated with the absence of inspiratory effort throughout the entire duration of the event and at least one of the criteria established in the AASM guidelines (the event lasts ≥20 s, the event lasted at least the duration of two breaths during baseline breathing and was associated with an arousal or a ≥3 % arterial oxygen desaturation and the event lasted at least the duration of two breaths during baseline breathing and was associated with a decrease in heart rate to less than 50 beats per minute (bpm) for at least 5 s). Mixed apnea was defined as meeting apnea criteria for at least the duration of 2 breaths during baseline breathing and being associated with the absence of respiratory effort during one part of the event and the presence of inspiratory effort during another part, regardless of which part came first.

Hypopnea was considered when all the following criteria were met: Peak signal excursion decreased by ≥ 30 % of the pre-event nasal pressure baseline, the duration of the ≥30 % decreased in signal excursion lasted for ≥2 breaths and there was a ≥3 % oxygen desaturation from the pre-event baseline or the event was associated with an arousal. Obstructive hypopneas were scored if any of the following criteria were present: There was snoring during the event, there was an increase in inspiratory flattening of the nasal pressure flow signal compared with baseline breathing or there was an associated thoracoabdominal paradox that occurred during the event but not during the pre-event breathing. Central hypopnea was considered if none of the previous criteria for obstructive hypopnea were met [16].

Oxygen saturation (SpO2) was interpreted based on the changes compared to the value obtained during wakefulness and the stable baseline values before each respiratory event. The desaturation index (DI) was defined as the number of decreases in SpO2>3 % per hour of the TTS. T90 and T85: percentage of TTS with SpO2 <90 and 85 % respectively.

OSA was defined from the polysomnographic point of view, as the presence of an Obstructive Apnea-Hypopnea Index (AHI) > 1/hour and classified according to severity as mild (2–5/hour), moderate (>5 to <10/hour) and severe (≥10/hour) using these three groups for analysis [16]. Although there are studies in children living at high altitude aged 3–5 years [17] and in children aged 7–14 years [18] that have shown that the normal values of sleep parameters differ from those of children at sea level due to higher total and central AHI and lower saturation, it is also important to note that the current evidence comes from prospective cross-sectional studies and some cohorts with small sample sizes that have shown variations between different age groups. Although the study by Ucros et al. sought to determine normal polygraph values in children aged 4–9 years in the city of Chiquinquira at 2560 m above sea level with a total population of 32 children [19], this study has some limitations such as small sample size and the use of polygraph instead of PSG. These limitations prevent the generalization of its results and the replacement of the criteria established by the ASSM For this reason, we have chosen to use the values established by the American Academy of Sleep Medicine (AASM).

### 2.2 Medical consultation

Referred patients underwent a medical evaluation, which included a medical history and a complete physical examination, including weight, height, blood pressure, oropharynx examination, and rhinoscopy. Body mass index (BMI) and its standard deviation were calculated, and the presence of overweight or obesity was determined according to World Health Organization (WHO) standards. Any child with a BMI >1 to ≤2 standard deviations (SD) was classified as overweight and any child with a BMI >2 SD was classified as obese according to the WHO classification [20].

### 2.3 Echocardiography

PH in children is defined as a mPAP greater than 25 mmHg measured by right heart catheterization [21,22]. TTE measurements can be used as a noninvasive screening test for PH. Although the article by Pang et al. estimated mPAP using a multiple regression equation, systolic pulmonary artery pressure via peak tricuspid regurgitation velocity (TRV) is currently used and is considered a surrogate measure of PH and is the echocardiographic variable recommended by the American Heart Association and American Thoracic Society guidelines for pulmonary hypertension screening in children [13,22].Transthoracic M-mode two-dimensional color doppler echocardiography was performed to determine the presence of PH according to the American College of Cardiology protocols using Vivid S6 General Electric echocardiography equipment. The TTE was performed by two pediatric cardiologists. Data included right ventricular outflow tract, aortic root, left atrial diameter, left ventricular outflow tract, interventricular septal thickness, left ventricular diameter at end diastole, left ventricular diameter at end systole, left ventricular posterior wall thickness, right ventricular diameter, right ventricular anterior wall thickness, pulmonary artery diameter, left ventricular ejection fraction and measurement of the TRV, if present, to determine pulmonary artery systolic pressure (PASP).

Estimation of PASP is based on the TRV using Bernoulli's equation. However, unlike in adults where the probability of pulmonary hypertension is determined from the TRV value [23], no studies have determined the normal values of PASP in children. Since there are currently no studies that have determined the probability of pulmonary hypertension from PASP, in consensus with the research group and taking into account the parameters used in other studies and reference values extrapolated from the adult population, the upper limit of normal (ULN) for right ventricular systolic pressure was set between 30 and 35 mmHg and >40 mmHg for the TTE diagnosis of PH([11,[24], [25], [26]]).

### 2.4 Statistical analysis

The information was collected using an instrument designed and adapted after a pilot test. It was then tabulated in Excel (version 2007) and exported to Stata 9.2 for analysis. Descriptive analysis was performed using frequency measures for qualitative variables and median and interquartile range (IQR) for quantitative variables due to the non-normal distribution of the variables. For univariate analysis, the chi-squared test (with Yates correction when appropriate) was used for categorical variables, and the Kruskall-Wallis test was used for differences between medians.

## 3 Results

**Fig. 1:**
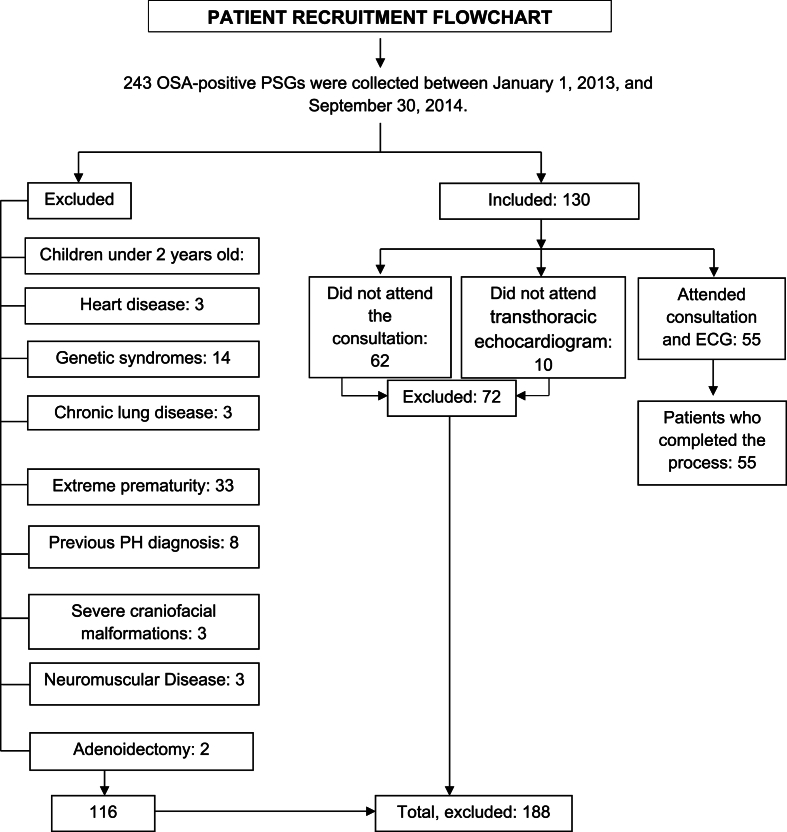
Patient recruitment flowchart.

A total of 243 PSGs with a diagnosis of OSA were collected, of which 130 met the inclusion criteria, 62 children did not attend the medical consultation and 10 did not attend the transthoracic echocardiography; a total of 55 patients were included. Fig. 1 shows patient recruitment. Of the 55 patients (n: 55), 63.6 % were male, with a median age of 6 years, 14 children (25.5 %) were overweight, 6 children (10.9 %) were obese, and 1 (1.8 %) was morbidly obese. When the severity of OSA was evaluated, 12 children (21.8 %) had mild OSA, 12 (21.8 %) had moderate OSA and 31 children (56.4 %) had severe OSA. Of all the patients studied, 17 children (31 %) had previous otolaryngologic evaluation and diagnosis of adenoid hypertrophy at the time of consultation without surgical treatment. 45.4 % of the patients had allergic rhinitis and/or asthma as associated comorbidity. The median time since the onset of symptoms suggestive of OSA was 2 years. The general characteristics of the population are shown in Table 1.

**Table 1:** Characteristics of the study population.

	Median (IQR)
Age, years	6 [[4], [5], [6], [7], [8]]
Male sex, n (%)	35 (63.6)
Weight(kg)	21.5 (16–33.5)
Size (cm)	112 (103–131)
BMI (Kg/M^2^)	16.9 (15.3–19.3)

Adenoidal hypertrophy, n (%)	17 [31]
Time of symptoms in years	2 [[1], [2], [3], [4]]
OSA severity, n (%) - Mild - Moderate - Severe	12 (21.8) 12 (21.8) 31 (56.4)
PASP values in the upper limit of normality (30–35 mmHg)	4 (7.3)

Abbreviations: BMI: body mass index; IQR: interquartile range; SD: standard deviation; OSA: obstructive sleep apnea. PASP: Pulmonary Artery Systolic Pressure. ULN: Upper Limit of Normality.

In children with OSA, the median AHI was 11 mostly due to obstructive events. Mean saturation during wakefulness was normal, with median saturation in REM and NREM sleep between 92 and 93 %. The median desaturation index was 23.8 (IQR: 13.8–37.7) (see Table 2). BMI was higher in patients with severe OSA, with a statistically significant association. The minimum saturation during events was lower in the severe OSA group (78 % (p = 0.013)) with a DI of 33.8/hour, similarly the mean saturation decreased with increasing severity (p < 0.05). T90 and T85 increased proportionally with OSA severity and were statistically significant (p < 0.05), the data are summarized in Table 3.

**Table 2:** Polysomnographic findings in children with OSA.

	Median (IQR)	Range
AHI	11 [[6], [7], [8], [9], [10], [11], [12], [13], [14], [15], [16], [17], [18], [19], [20], [21], [22], [23], [24]]	2.4–85
CAHI	1.9 (1.2–5.6)	0–27.7
OAHI	7 [[4], [5], [6], [7], [8], [9], [10], [11], [12], [13], [14], [15]]	0.3–82.7
SpO_2_ in wakefulness, %	94 (93–95)	89–96
SpO_2_ in REM sleep, %	92 (90–94)	78–96
SpO_2_ in non-REM sleep, %	93 (92–93)	84–95
Average SpO_2_ in events, %	89 (86–90)	61–93
Minimum SpO_2_ in events, %	79 (74–84)	42–90
T90, %	2.8 (0.6–15)	0–78
T85, %	0.1 (0.02–1.3)	0–29
ODI	23.8 (13.8–37.7)	4.9–128.2

Abbreviations: IQR: Interquartile Range; AHI: apnea-hypopnea index; OAHI: Obstructive Apnea-Hypopnea Index; CAHI Central Apnea-Hypopnea Index; SpO_2_: Oxygen Saturation; REM: Rapid Eye Movement; T90: Percentage of total sleep time with SpO_2_ below 90 %; T85: Percentage of total sleep time with SpO_2_ below 85 %; ODI: Oxygen Desaturation Index (number of desaturations/sleep hour).

**Table 3:** Polysomnographic characteristics according to OSA severity and BMI.

	Mild OSA (AHI:2–5/h) Median (IQR)	Moderate OSA (AHI:>5–10/h) Median (IQR)	Severe OSA (AHI>10/h) Median (IQR)	P value
BMI	16 (14.7–17.3)	16.8 (16–17.3)	18.2 (15–20.5)	0.22
BMI, SD	−0.45 (−0.96-0.11)	0.69 (0.06–1.34)	1.2 (−0.14-2)	0.035
SpO_2_ in wakefulness, %	94 (93–94.5)	95 (94–95)	94 (93–95)	0.24
SpO_2_ in REM sleep, %	93 (92–94)	93.5 (92–94.5)	92 (88–93)	0.043
SpO_2_ in non-REM sleep, %	93 (92–93)	93.5 (93–94)	92 (91–93)	0.015
Average SpO_2_ in events, %	89 (87–90)	89 (88–91)	88 (83–89)	0.11
Minimum SpO_2_ in events, %	85.5 (78–86.5)	80.5 (76.5–84.5)	78 (66–82)	0.013
T90, %	1.45 (0.32–5.5)	0.95 (0.25–2.5)	9 (1.5–17)	0.0096
T85, %	0.01 (0–0.185)	0.04 (0–0.095)	1 (0.06–4)	0.0004
ODI	11.8 (9.7–20.6)	16.2 (12.3–20.6)	33.8 (23.8–62.5)	0.0001

Abbreviations: IQR: Interquartile Range; AHI: apnea-hypopnea index; OAHI: Obstructive Apnea-Hypopnea Index; CAHI Central Apnea-Hypopnea Index; SpO_2_: Oxygen Saturation; REM: Rapid Eye Movement; T90: Percentage of total sleep time with SpO_2_ below 90 %; T85: Percentage of total sleep time with SpO_2_ below 85 %; ODI: Oxygen Desaturation Index (number of desaturations/sleep hour).

Of the 55 patients with OSA, none had PH according to TTE; 4 patients (7.2 %) had PASP at the upper normal limit for their age (between 30 and 35 mmHg), and it was not associated with higher BMI or longer time to OSA symptoms. Patients with PASP at the upper normal limit had lower minimum and mean saturations than those with PASP in lower ranges, but this was not statistically significant. Regarding OSA severity, 2 of the 4 children had severe OSA and the other 2 had mild OSA, without statistical significance (see Table 4).

**Table 4:** Comparison between OSA severity, BMI, SpO_2_, and PASP.

	PASP in ULN Median (IQR) n = 4	PASP below ULN, Median (IQR) n = 51.	P value
Time of symptoms in years	1.5 [[1], [2], [3], [4], [5]]	2 (1.5–4)	0.09
BMI	17.5 (15.7–19.6)	16.9 (15–19.1)	0.87
BMI, SD	−0.005 (−0.76 - 0.785)	0.68 (−0.39 - 1.96)	0.31
Average SpO_2_, %	86.5 (82.5–89)	89 (87–90)	0.36
Minimum SpO_2_, %	78 (71.5–78.5)	80 (74–84)	0.29
OSA severity, n (%)			
Mild	2(50 %)	10 (19.6 %)	
Moderate	0	12 (23.5 %)	
Severe	2 (50 %)	29 (56.9 %)	0.28

*Abbreviations: BMI: body mass index; IQR: interquartile range; BMI: body mass index; SD: standard deviation; OSA: obstructive sleep apnea. PASP: Pulmonary Artery Systolic pressure. ULN: Upper Limit of Normality; SpO*_*2*_*: Oxygen Saturation*.

## 4 Discussion

This is the first cross-sectional study focused on children living permanently at HA (2640 m) in a tropical country with obstructive sleep apnea to determine whether living at high altitude is an additional risk factor for the development of PH in children with OSA. However, contrary to our expectations, we found no evidence of PH in the 55 consecutively diagnosed children with OSA. Only 4 of these children (7.3 %) had PASP that was within the upper limits of normal values. These unexpected findings raise important questions about the possible mechanisms underlying the negative results.

Before discussing the possible implications of the present results, some methodological considerations should be mentioned. First, our PASP cut-off point for the echocardiographic diagnosis of pulmonary hypertension was strict (PASP >40 mmHg), taking into account the study by Vasquez et al. performed in Mexico City at 2240 m (moderate altitude), where a cut-off point of 32 mmHg was used and a prevalence of 7 % was found in a sample of 158 children [27]; a prevalence that would be similar in our study if we used the same cut-off point. However, having a lower cut-off point decreases specificity and increases the risk of overdiagnosis and of studying patients without indication, especially when living at high altitudes, considering that, according to the studies in adults, a PASP of <35 mmHg implies a TRV of less than 2.8, which confers a low probability of pulmonary hypertension [23,28,29].

It is important to note that the normal values for pulmonary artery pressure in children are not standardized. McQuillan et al. [30], in their study of normal values describe an upper normal limit of PASP of 36 mmHg in males and 34 mmHg in females for the population under 20 years of age (856 subjects), Berger et al. [31] establishes values of 32 and 30 mmHg for males and females, respectively and the study by Pang et al. used a multiple regression equation to estimate the mPAP and did not calculate PASP(13). This variability of cut-off points at sea level, and the absence of normality parameters at high altitudes explain the differences found and the difficulty of conducting research in this area [[32], [33], [34]].

It is also important to emphasize that like what has been noted regarding the cut-off point for PASP at high altitude, the same is true for the AHI values; currently there are few studies in healthy children that establish normal values in school children, and the existing literature suggests that respiratory disorders during sleep are more common at high altitude. A previous study of ours and the study by Hill et al. show an improvement of these parameters with age, from infancy to childhood, suggesting a developmental adaptation of respiratory sleep physiology at high altitude [35,36]. The systematic review by Ucros et al. on the respiratory pattern during sleep at high altitude included mostly infants and only two studies in schoolchildren, one of which has a small number of patients and was performed with polygraphy, and the other by Hill et al. in schoolchildren and adolescents, in which they reported IAHO values of 2(3.5) and IAO: 0(0) [37]. Recently, our group established normal values for children aged 5–17 years and found a median IAHO of 1.4(0.9–2.1) in children older than 10 years and 0–9(0.5–1.3) in children younger than 10 years [38].

The most important finding of our study is the absence of PH in children with different degrees of OSA living at high altitude, with minimal SaO2 during events of 85 % or less, with elevated T90, T85, and ID, suggesting the presence of intermittent hypoxia.

The association between OSA and the cardiovascular complications of this entity is clear in the adult population; however, in children, this association is not fully defined, and few studies document it. The old studies available on the clinical and hemodynamic characteristics of children with OSA and pulmonary hypertension and cor pulmonale are limited [16,[39], [40], [41], [42], [43]], with small numbers of patients and polysomnographic and echocardiographic techniques different from those currently used, most of them did not establish the presence of intermittent hypoxia, nor did they evaluate oxygenation parameters and some were performed without polysomnographic recordings, based only on clinical diagnosis, making it difficult to compare them with current studies.

Recent studies on the prevalence of PH in children with OSA show variable results, mostly with findings similar to ours with a low prevalence of PH in this group of children; similar to our results, the studies by Clements et al. [44] and Teplitzky et al. [45] found no association between the degree of severity of OSA and abnormal echocardiographic findings. Similarly, a systematic review of echocardiographic findings in children with obstructive sleep apnea in the pediatric population found no consistent results; of the 13 studies that met the inclusion criteria, only 2 measured the TRV, and of these, only one found a positive correlation with AHI([46]).

In contrast to the above, in a systematic review and meta-analysis by Weber et al. [47] found that that patients with OSA had a statistically significant increase in interventricular septal thickness and right ventricular dimension, which improved after adenotonsillectomy. In this study, the prevalence of PH in children with OSA at high altitude is low and similar to current studies such as Burns et al. [10] and Birtnes et al. [48] at sea level who found a prevalence of 1.8 % and 4 %, respectively, as mentioned at the beginning, if our study had established cut-off points similar to those at sea level, our prevalence would have been higher.

Ingram et al. [49] reported variable prevalence from 0 to 85 % of pulmonary hypertension in OSA and from 6 % to 24 % of OSA in pulmonary hypertension, Maloney et al. [11] found a prevalence of 8 % in their study of 318 children with severe obstructive apnea, but this study included children with Down syndrome (46 %) who have other causes or factors associated with PH.

Nowadays, the early diagnosis of OSA in children may explain the lack of prevalence of PH in our high-altitude population, and it is also possible that the adaptive phenomenon of chronic hypoxia is a protective factor for triggering PH.

Multiple factors play a role in the morbidity associated with pediatric OSA. The severity, and magnitude of the inflammatory response to oxidative stress, individual and genetic susceptibility factors, and environmental modifiers may all exacerbate or attenuate the degree of organ damage leading to the presence of OSA in children [50]. In addition, it is possible that exposure to recurrent hypoxic events during childhood may cause the pulmonary circulation to respond differently to vasoconstrictor stimuli in the future, and that the pulmonary vascular response due to hypoxia may be exaggerated in adulthood, leading to pulmonary hypertension.

Even in adults, there are wide variations in the literature regarding the prevalence of PH in patients with OSA, which may be related to environmental factors, genetic predisposition, obesity, or left ventricular dysfunction [51]. Cai et al. [52] found that children with OSA had a thicker right ventricular anterior wall, smaller right ventricular diameter, and wider pulmonary arteries, suggesting increased pulmonary artery pressure. However, we did not find these findings in our study; only one patient had left ventricular hypertrophy, without any other cardiac remodeling changes on echocardiogram. Similar findings were observed by Amin et al. [53] who demonstrated a dose-dependent increase in left ventricular mass index with increasing severity of OSA. In a later study by the same author, an independent effect of OSA on left ventricular function was observed in children, and a negative correlation between the severity of OSA and left ventricular diastolic function [54]. Due to the absence of PH in our patients, we cannot establish a relationship between the left ventricular findings and pulmonary hypertension.

The results of this study are consistent with others regarding the low prevalence of pulmonary hypertension in OSA in patients without other comorbidities. Ingram et al. recommend performing a polysomnogram in all children with newly diagnosed pulmonary hypertension and performing an echocardiogram in children with OSA of any severity associated with any comorbidity [49]. We suggest individualizing each case. Although pediatric patients with OSA seem to have a very low prevalence of PH, it is ideal to confirm this finding in a longitudinal study with multiple measurements over time in the groups of patients at the highest risk.

Our study is innovative in showing that children with OSA living in a high-altitude tropical city have a low prevalence of PH. These findings may be applicable to the nearly 400 million people worldwide who live permanently above 1500 m [55]. This study is also innovative in terms of the PASP cut-off point used to determine the likelihood of pulmonary hypertension, because as mentioned in the other studies conducted, there is a high variability in this aspect. Although the study focused on children living at high altitude, the results contribute to the scarce information on OSA and PH in Latin America [56].

Some limitations include the lack of a control group residing at low altitude due to the economic and logistical difficulties of assembling it in our country and the small sample size. Two other limitations of our study are the use of normal values of PSG at sea level in the absence of standardized values at high altitude for the reasons mentioned above, and the fact that our population included children between 2 and 16 years of age. This is important considering that in the meta-analysis by Uros et al. 6 of the included studies were performed in infants [37], which were not part of our population; the study by Uros et al. included only children up to 9 years of age and used polygraphy instead of PSG(19), and the study by Hill et al. included children from 9 years of age [57]; therefore, extrapolation of the data from these two studies to our population was not considered appropriate from a methodological point of view. In addition, the use of PASP values reported at sea level is also a limitation; however, as noted above, there is no consensus on this issue, and the study by Pang et al., although using echocardiography, did not report PASP(13), which is based on the TRV, the variable recommended for adults and children at as a screening tool to identify patients with possible pulmonary hypertension [22,58]. This study determined mPAP values by a multiple regression equation based on echocardiographic values and also reported the values of right ventricular systolic time interval (RSTI), previous ejection period (PEP), ascent time (AT), ejection time (ET), PEP/AT, and AT/ET variables [13], which are not recommended for PH screening according to reference guidelines [22], so we did not use these variables in our study.

Finally, all children were evaluated by radiologic and/or endoscopic examination to determine the presence of adenoid hypertrophy. Unfortunately, since this was a retrospective study based on a review of medical records, information on the size of the adenoids was not recorded.

## 5 Conclusion

In the present study, it was not possible to establish the association between OSA and PH in pediatric age in a high-altitude city. However, because OSA is an increasingly recognized condition in children and is known to cause cardiovascular complications, current guidelines recommend screening for PH in patients with OSA, but OSA severity, intermittent desaturation, and altitude do not appear to be markers for determining the risk of developing PH. Given the low prevalence of pulmonary hypertension in children with OSA even in those living at high altitude, further studies are needed to determine which subgroups of OSA may benefit from screening for pulmonary hypertension.

## Data availability statement

The data on which this article is based are available through the Research Department of the Fundación Neumológica Colombiana and can be requested from the authors of this article as well as from the Research Department of the Fundación Neumológica Colombiana.

## Funding

This study did not receive any funding

## CRediT authorship contribution statement

**Elida Duenas-Meza:** Writing – review & editing, Writing – original draft, Supervision, Resources, Project administration, Methodology, Investigation, Formal analysis, Data curation, Conceptualization. **Diego Fernando Severiche-Bueno:** Writing – review & editing, Writing – original draft, Visualization, Validation, Investigation, Formal analysis, Conceptualization. **Carolina Santos Quintero:** Validation, Supervision, Methodology, Investigation, Conceptualization. **Jenny Talani Ochoa:** Methodology, Investigation, Formal analysis, Data curation, Conceptualization. **Miguel Ronderos Dummit:** Resources, Investigation, Formal analysis, Conceptualization. **Claudia Stapper:** Resources, Methodology, Formal analysis, Conceptualization. **Carlos Granados G:** Resources, Methodology, Investigation, Conceptualization.

## Declaration of generative AI and AI-assisted technologies in the writing process

During the preparation of this work the author(s) used DeepL Write in order to improve readability. After using this tool/service, the author(s) reviewed and edited the content as needed and take(s) full responsibility for the content of the publication.

## Declaration of competing interest

The authors declare that they have no known competing financial interests or personal relationships that could have appeared to influence the work reported in this paper.
